# 单操作孔全腔镜肺癌根治术113例分析

**DOI:** 10.3779/j.issn.1009-3419.2014.05.11

**Published:** 2014-05-20

**Authors:** 春 徐, 海涛 马, 斌 倪, 靖康 何, 畅 李, 成 丁, 广斌 李, 宇轩 王, 军 赵

**Affiliations:** 215006 苏州，苏州大学附属第一医院心胸外科 Department of Thoracic and Cardiovascular Surgery, the First Affiliated Hospital of Soochow University, Suzhou 215006, China

**Keywords:** 单操作孔, 肺叶切除肺癌根治术, 肺肿瘤, Single-operation-hole, Lobectomy in lung cancer resection, Lung neoplasms

## Abstract

**背景与目的:**

胸腔镜肺叶切除术治疗肺癌已经被广泛接受，本研究探讨单操作孔全腔镜下非小细胞肺癌根治术的可行性。

**方法:**

回顾性研究分析本院2010年10月至2013年10月共为113例非小细胞肺癌患者施行单操作孔全腔镜肺癌根治术。胸腔镜观察孔取腋中线后侧第8肋间，切口约1.5 cm，操作孔取腋前线第4或5肋间，切口长约2 cm-4 cm，经单一操作孔完成胸腔内手术操作。

**结果:**

全组患者手术顺利，无围手术期死亡，其中5例患者因术中大出血行操作孔撑开；平均手术时间（178.24±31.37）min，平均术中失血（213.56±62.38）mL，术中清扫淋巴结5枚-22枚。3例患者因术后并发症再次行胸腔镜下手术，其中2例为迟发性出血，1例为乳糜胸。全组患者术后病理均证实肺癌诊断，术后平均住院时间（8.17±2.93）d。术后患者均顺利恢复，随访2个月-38个月仅5例出现复发或转移。

**结论:**

单操作孔全腔镜肺癌根治术安全可行，进一步降低了创伤，可以作为早中期非小细胞肺癌的一种常规手术方式。

## Abstract (en)

**Background and objective:**

Video-assisted thoracoscopic surgery (VATS) lobectomy is generally accepted for patients with lung cancer. The aim of this study is to explore the feasibility of the single-operation-hole thoracoscopic lobectomy in the treatment of non-small cell lung cancer.

**Methods:**

To review and analyze the single-operation-hole thoracoscopic lobectomy performed in our hospital for 113 non-small cell lung cancer (NSCLC) cases from October 2010 to October 2013. The incision for observation was 1.5 cm the eighth intercostal at the rear of the midaxillary line and the incision for operation was 2.0 cm-4.0 cm at the fourth or fifth intercostal of the anterior axillary line. The operations were performed through the single-operation-hole.

**Result:**

The operation processes were smooth for all the patients without any operative mortality occurrence. Only in 5 cases was the operation hole expanded because of the occurrence of massive hemorrhage during the operation; 3 patients with postoperative complications underwent thoracoscopic lobectomy again, including 2 cases of delayed hemorrhage and 1 case of chylothorax. The average surgical duration was (178.24±31.17) min, the average blood loss was (213.56±62.38) mL, and the number of lymph nodes dissected was from 5-22. All diagnose were confirmed by pathology after operation. The average length of stay was (8.17±2.93) d. All cases recovered well during the follow-up of (2-38) months, only 5 cases had recurrence or metastasis.

**Conclusion:**

The single-operation-hole thomcoscopic lobectomy for lung cancer is safe and feasible, further reducing the trauma, and can be used as a conventional treatment for early- or medium-term NSCLC..

近年来随着微创概念的深入人心和胸腔镜技术的日益发展，胸腔镜肺叶切除术应用于肺癌治疗已逐渐被广泛接受，已成为胸外科领域最受关注的手术技术之一。胸腔镜肺叶切除肺癌根治手术与传统开胸手术相比，可明显减轻全身炎症反应、减少围手术期并发症、缩短术后胸管引流时间及住院时间，具有较好的疗效^[1-3]^。在此基础上，我单位于2010年10月-2013年10月对113例周围型肺癌患者进行了单操作孔全腔镜肺癌根治术，为微创胸外科手术在肺癌治疗的进一步应用做了尝试，取得了较好临床效果，现总结报告如下。

## 1 资料与方法

### 1.1 临床资料

全组113例，男69例，女44例，年龄33岁-82岁，中位年龄63.2岁；无吸烟史31例，吸烟史 < 5年11例，5-10年24例， > 10年47例；所有患者术前未行放化疗，术前辅助检查排除转移性病变存在，无严重合并症、可耐受手术；病变位于左肺上叶20例，左肺下叶31例，右肺上叶17例，右肺中叶11例，右肺下叶34例；术前通过纤维支气管镜和CT引导下穿刺活检明确病理学诊断有34例，其中鳞癌14例，腺癌20例，术中通过快速冰冻切片明确病理后行肺癌根治术79例，其中先将肿瘤楔形切除51例，直接肺叶切除28例。

### 1.2 单操作孔胸腔镜肺癌根治术的适应证

按以下条件对有手术指征的非小细胞肺癌患者进行选择，同时符合下列条件者实行单孔胸腔镜手术治疗：①肿瘤直径小于7 cm；②纤维支气管镜检查肿瘤未累及叶支气管开口；③既往无术侧胸部手术史，胸腔内无严重致密粘连；④术前增强CT提示淋巴结无成串钙化融合；⑤患者及其家属有强烈的微创手术要求。

### 1.3 手术方法

采用双腔气管插管全身麻醉，单肺通气；患者取健侧卧位，肩下垫软枕，手术床摇成折刀位以增加肋间隙宽度。术者位于患者腹侧，由腋中线偏后第8肋间作长1.5 cm切口置入30°腔镜作为观察孔，于腋前线第4或第5肋间作2 cm-4 cm切口作为操作孔，孔内放置橡胶弹性切口保护套，不放置撑开器，仅供器械进出使用。所有操作均在胸腔镜下完成，使用电凝钩结合双关节专用腔镜器械帮助完成镜下分离、切开等操作，必要时使用超声刀进行淋巴结清扫。

肺叶切除一般先行游离肺门结构，但处理顺序无固定模式。一般先经肺门前方或下肺韧带游离、切断肺静脉，后处理叶支气管，再分支处理肺动脉，最后处理不全叶裂。肺叶切除后一律进行系统性肺门和纵隔淋巴结清扫，右侧包括2、3、4、7、8、9、10组淋巴结，左侧包括5、6、7、8、9、10组淋巴结。肺静脉、支气管、部分肺动脉、分化不全的肺裂处理使用内镜直线切割缝合器，肺动脉部分分支处理亦可使用丝线结扎、Hamolock或可吸收血管夹夹闭近、远端后切断。

### 1.4 统计学分析

采用SPSS 20.0软件进行统计分析，计量资料采用*t*检验，计数资料采用卡方检验。

## 2 结果

全组患者手术顺利，无围手术期死亡，行左上肺叶切除术20例，左下肺叶切除术31例，右上肺叶切除术17例，右中肺叶切除术11例，右下肺叶切除术34例。全组手术时间（70-285）min，平均（178.24±31.17）min。术中失血50 mL-700 mL，平均（213.56±62.38）mL。术中清扫淋巴结5枚-24枚，平均（11.51±3.63）枚。术后留置胸管时间1 d-11 d，平均（3.39±1.72）d。术后引流量150 mL-2, 000 mL，平均（573.65±277.48）mL。术后住院时间3 d-16 d，平均（8.17±2.93）d。5例患者因术中突发出血行操作孔撑开辅助手术。围手术期并发症主要有余肺肺不张10例（8.8%）、余肺感染5例（4.4%）、迟发性出血2例（1.8%）、乳糜胸1例（0.9%），其中2例迟发性出血和1例乳糜胸患者再次行腔镜下手术治疗。全组患者术后病理均证实肺癌诊断，其中鳞癌39例，腺癌60例，腺鳞癌5例，肺泡细胞癌7例，大细胞癌2例；病理分期为Ⅰa期（T1N0M0）46例，Ⅰb期（T2aN0M0）29例，Ⅱa期21例（T1N1M0, T2aN1M0, T2bN0M0），Ⅱb期6例（T2bN1M0），Ⅲa期11例（T1N2M0, T2N2M0）。全组术后均随访2个月-38个月，2例患者出现同侧肺内复发，2例患者出现颅内转移，1例患者出现骨转移。

## 3 讨论

在微创外科技术飞跃发展的今天，全腔镜肺叶切除术治疗肺癌因其可行性及优越性已逐渐被大多数胸外科医师所接受。在不违反肿瘤治疗标准及胸部手术切除基本原则的情况下，选择全腔镜下肺癌根治术来治疗非小细胞肺癌是合理的。但目前对全腔镜肺叶切除术操作孔的选择尚有争议，最常用包括“三孔法”和“四孔法”^[1, 4, 5]^。我单位在不断积累全腔镜肺叶切除手术经验的基础上，采用“双孔法”，即除观察孔外仅通过单一操作孔完成全腔镜肺癌根治手术，取得了一定经验和明显疗效。

单操作孔全腔镜肺癌根治术医源性创伤更小，避免了增加其余副操作孔带来的肌肉神经损伤，进一步减少了切口出血的概率，从而减少了止血带来的额外手术时间，术后疼痛的程度也大大降低。对于女性患者，单操作孔可做于乳房下缘，术后胸部一般仅显露观察孔一处手术疤痕，较其它术式更具有良好的美学效果。概括而言，单操作孔全腔镜肺癌根治术具有“视野清、切口短、损伤小、耗时少、疼痛轻、活动早、恢复快、出院早”等优势。

术者是单操作孔胸腔镜肺癌根治术成功与否的关键。术者首先要有一定耐心和多指协调性，得熟练掌握传统的开胸手术技巧，能应对术中被迫中转开胸等意外状况，能单孔熟练使用数把腔镜器械对组织和血管进行暴露、游离、结扎等操作；其次，术者对镜下肺叶解剖结构的二维视野要有精确的方向感知，这样可以避免确认解剖结构时带来的不必要的肺叶翻动，减少手术耗时，避免操作误伤。除了对术者的技巧要求外，患者的病理诊断也影响着单操作孔胸腔镜肺癌根治术的方式。患者术前若肺癌病理诊断明确，则直接行肺癌根治术；否则，术中一般先通过操作孔探查，明确病变位置，周围型病变先行肺楔形切除，送快速病理明确肺癌诊断后行肺癌根治术；若探查见肿瘤胸膜牵拉征明显，结合术前PET-CT等检查提示肿瘤恶性证据充分，或肿瘤较大靠近肺门无法楔形切除，则直接先行肺叶切除后再送快速冰冻切片。所有病例淋巴结清扫均在病理明确肺癌诊断的基础上进行。

全组病例术中观察孔选择第8肋间腋中线和腋后线之间肋间隙相对较宽处，便于观察支气管位置及术后放置引流；根据不同肺叶肿瘤的位置以及叶裂与胸壁位置关系选择腋前线第4或第5肋间为操作孔，便于肺门血管尤其是动脉分支处理，一般情况下上叶肿瘤选择第4肋间、中叶和下叶肿瘤选择第5肋间。传统胸腔镜肺叶切除术常规采取肺静脉→叶间裂→肺动脉→支气管的操作步骤^[6]^；刘伦旭等^[7]^提出单向式切除方法，主张肺门操作按肺静脉→支气管→肺动脉→叶间裂的游离顺序。我们的经验表明，单向式的切除方法更易操作，本组病例在肺静脉切断之后一般选择先行处理叶支气管，此法优点在于叶支气管一般位置较为固定，处理后牵拉暴露肺门活动度更大，从而部分抵消了单孔操作对暴露的不利影响，方便后续动脉分支及不全叶裂的处理。某些叶支气管或动脉分支由于角度特殊，游离完成后需临时将观察孔改为操作孔，腔镜则由前操作孔置入，在镜下引导一次性切割闭合器自原观察孔置入，此法可有效避免常规路径下因切割器与需处理结构平行而无法合理操作的问题，缩短手术时间。同时我们将传统方式中的观察孔由腋中线移至靠近腋后线，也方便了对肺门结构的处理。对于支气管、静脉、较粗动脉，我们常规予切割闭合器切断，动脉较细分支则予结扎、上血管闭合夹（Hemolock）处理后切断。我们的经验是血管游离长度应尽量长，以免切割缝合器置入困难，甚至撕裂血管分叉导致出血。本组病例选择前操作孔靠近叶裂的第4或第5肋间，在处理动脉分支及不全叶裂时暴露更为充分，有利操作。对发育不全肺裂内较细的动脉分支难以剥离时，强行分离易造成肺组织损伤较多，术后漏气时间延长，本组中尝试采用一次性缝合切开器一并处理肺裂及其中细小血管分支，效果良好，均未出现出血、漏气等相关并发症。

由于系统性淋巴结清扫可以更好的控制肿瘤局部复发，且不增加围手术期并发症发生率和病死率^[8]^，本组病例术中常规进行肺门及纵隔淋巴结清扫。文献报道胸腔镜下淋巴结清扫由于其视野清楚，操作无“盲区”，可以达到与传统开胸手术相同的效果^[6, 8]^，但由于镜下操作的特点，我们总结了如下经验：清扫上纵隔淋巴结时，应注意避免损伤神经，尽量少用电凝钩或超声刀，多采用钝性及锐性分离，取出淋巴结后再用纱布压迫，谨慎止血；隆突下淋巴结右侧较左侧表浅，相对易暴露，在下叶切除之前清扫，位置相对固定更容易，同时可留出更充裕的时间观察淋巴结床有无渗血，彻底清扫完成后可见隆突尖部、左右主支气管、食管壁、心包。清扫第10、11组淋巴结时，打开血管鞘清扫是最有效的方法。对增大明显、有外侵的淋巴结分离应电凝结合超声刀，仔细辨认胸导管、支气管动脉等重要结构后分离淋巴结并不增加手术时间，能减少误伤及术后引流量。进行系统性淋巴结清扫后胸腔引流液可能会增加，这可能与毛细淋巴管瘘有关，全组病例有1例右上肺癌患者术后第二天出现乳糜胸，保守治疗两天无改善，胸液引流大于1, 000 mL/d，再次胸腔镜下行胸导管结扎术。

应对单操作孔胸腔镜肺癌根治术中遇到的一些特殊情况，我们主要有以下经验：对于胸腔粘连，由于胸腔镜的多角度及放大作用，整个胸腔不存在死角，术者对创面出血的观察更为细致，处理亦更加精确。但对于胸腔弥漫致密粘连患者，纵隔面粘连的分离是较为困难的步骤，应耐心谨慎，强调在清楚暴露的前提下分离粘连，以防止上腔静脉、锁骨下动脉等重要结构损伤。严重粘连患者术后往往引流量偏多，住院时间相对延长。另外，如果肿瘤较大，则术中翻动或牵拉肺叶时困难大大增加，不利于术野暴露，我们则通过摇床来使肺组织自然下垂，增加手术操作空间；同时肿瘤较大也给标本取出带来一定困难，徐迟等^[9]^采用延长主操作孔将肿瘤取出，我们则将较大瘤体装入标本袋中剖开后取出，由于有标本袋保护，肿瘤细胞亦不会出现播散情况。如果术中遇到肺门淋巴结钙化融合致肺叶血管游离困难、镜下难以控制的出血等情况时，应果断撑开操作孔辅助手术。全组病例中有5例患者在术中解剖肺动脉分支及剥离淋巴结时出现难以控制的大出血，行操作孔撑开辅助操作。

全组病例施行单操作孔肺癌根治术后的并发症和其他术式基本类似，主要为余肺不张、余肺感染，术中减少对肺组织的反复钳夹及翻动，拔除气管插管前充分吸痰，术后加强护理、督促咳嗽排痰能有效减少此类并发症的发生。另有2例患者因迟发性出血行二次腔镜手术止血，原因是单操作孔全腔镜手术早期经验缺乏，右下肺叶气管残端生物胶涂抹太多，患者剧烈咳嗽后坚硬的残端与椎旁胸壁摩擦导致肋间动脉破裂出血。因此，术中切割气管时应注意避免气管残端与胸壁垂直，同时尽量少使用生物胶，或者可在气管残端对应胸壁面贴以明胶海绵来减少残端反复摩擦而导致的肋间动脉损伤。

综上所述，单操作孔全腔镜肺癌根治术作为一种新的手术方式，更符合微创胸外科的理念，为一部分经过选择的周围型肺癌患者提供了更小创伤、更少痛苦、更快恢复的治疗途径。随着胸外科医生腔镜操作技巧日益娴熟、腔镜设备的不断完善，同时经过不断总结经验，单操作孔全腔镜肺癌根治术定是一种有发展前途的方法，值得在临床中加以应用和推广。
